# Supplementary material for: A PON for All Seasons: Comparing Paraoxonase Enzyme Substrates, Activity and Action including the Role of PON3 in Health and Disease
Source: Antioxidants (Basel). 2022 Mar 19;11(3):590. doi: 10.3390/antiox11030590 (PMC8945423; doi:10.3390/antiox11030590)
Supplement: Supplementary file 1 [file antioxidants-11-00590-s001.zip › antioxidants-1632931-supplementary.pdf]

Supplemental Table S1. Predicted glycosylation patterns and catalytic regions of Paraoxonase isoforms.

Residue position	PON1 Amino Acid Abbreviation	PON1 Glycosylation prediction	PON2 Amino Acid Abbreviation	PON2 Glycosylation prediction	PON3 Amino Acid Abbreviation	PON3 Glycosylation prediction
1	M	-	M	-	M	-
2	A	-	G	-	G	-
3	K	-	R	-	K	-
4	L	-	L	-	L	-
5	I	-	V	-	V	-
6	A	-	A	-	A	-
7	L	-	V	-	L	-
8	T	n	G	-	V	-
9	L	-	L	-	L	-
10	L	-	L	-	L	-
11	G	-	G	-	G	-
12	M	-	I	-	V	-
13	G	-	A	-	G	-
14	L	-	L	-	L	-
15	A	-	A	-	S	n
16	L	-	L	-	L	-
17	F	-	L	-	V	-
18	R	-	G	-	G	-
19	N	n	E	-	E	-
20	H	-	R	-	M	-
21	Q	-	L	-	F	-
22	S	n	L	-	L	-
23	S	n	A	-	A	-
24	Y	-	L	-	F	-
25	Q	-	R	-	R	-
26	T	n	N	n	E	-
27	R	-	R	-	R	-
28	L	-	L	-	V	-
29	N	n	K	-	N	G (N[1])
30	A	-	A	-	A	-
31	L	-	S	n	S	n
32	R	-	R	-	R	-
33	E	-	E	-	E	-
34	V	-	V	-	V	-
35	Q	-	E	-	E	-
36	P	-	S	n	P	-

37	V	-	V	-	V	-
38	E	-	D	-	E	-
39	L	-	L	-	P	-
40	P	-	P	-	E	-
41	N	n	H	-	N	n
42	C	-	C	-	C	-
43	N	n	H	-	H	-
44	L	-	L	-	L	-
45	V	-	I	-	I	-
46	K	-	K	-	E	-
47	G	-	G	-	E	-
48	I	-	I	-	L	-
49	E	-	E	-	E	-
50	T	n	A	-	S	n
51	G	-	G	-	G	-
52	S	n	S	n	S	n
53	E*Cat	-	E*Cat	-	E*Cat	-
54	D*Cat	-	D*Cat	-	D*Cat	-
55	L	-	I	-	I	-
56	E	-	D	-	D	-
57	I	-	I	-	I	-
58	L	-	L	-	L	-
59	P	-	P	-	P	-
60	N	n	N	n	S	n
61	G	-	G	-	G	-
62	L	-	L	-	L	-
63	A	-	A	-	A	-
64	F	-	F	-	F	-
65	I	-	F	-	I	-
66	S	G	S	G	S	G
67	S	G	V	-	S	G
68	G	-	G	-	G	-
69	L	-	L	-	L	-
70	K	-	K	-	K	-
71	Y	-	F	-	Y	-
72	P	-	P	-	P	-
73	G	-	G	-	G	-
74	I	-	L	-	M	-
75	K	-	H	-	P	-
76	S	n	S	n	N	n
77	F	-	F	-	F	-

78	N	n	A	-	A	-
79	P	-	P	-	P	-
80	N	G	D	-	D	-
81	S	n	K	-	E	-
82	P	-	P	-	P	-
83	G	-	G	-	G	-
84	K	-	G	-	K	-
85	I	-	I	-	I	-
86	L	-	L	-	F	-
87	L	-	M	-	L	-
88	M	-	M	-	M	-
89	D	-	D	-	D	-
90	L	-	L	-	L	-
91	N	n	K	-	N	G
92	E	-	E	-	E	-
93	E	-	E	-	Q	-
94	D	-	K	-	N	n
95	P	-	P	-	P	-
96	T	G	R	-	R	-
97	V	-	A	-	A	-
98	L	-	R	-	Q	-
99	E	-	E	-	A	-
100	L	-	L	-	L	-
101	G	-	R	-	E	-
102	I	-	I	-	I	-
103	T	n	S	G	S	n
104	G	-	R	-	G	-
105	S	G	G	-	G	-
106	K	-	F	-	F	-
107	F	-	D	-	D	-
108	D	-	L	-	K	-
109	V	-	A	-	E	-
110	S	n	S	G	L	-
111	S	G	F	-	F	-
112	F	-	N	G	N	n
113	N	G	P	-	P	-
114	P	-	H	-	H	-
115	H	-	G	-	G	-
116	G	-	I*Cat	-	I*Cat	-
117	I*Cat	-	S	n	S	n
118	S	G	T	G	I	-

119	T	n	F	-	F	-
120	F	-	I	-	I	-
121	T	n	D	-	D	-
122	D	-	N	G	K	-
123	E	-	D	-	D	-
124	D	-	D	-	N	G
125	N	n	T	G	T	G
126	A	-	V	-	V	-
127	M	-	Y	-	Y	-
128	Y	-	L	-	L	-
129	L	-	F	-	Y	-
130	L	-	V	-	V	-
131	V	-	V	-	V	-
132	V	-	N	G	N	G
133	N	G	H	-	H	-
134	H	-	P	-	P	-
135	P	-	E	-	H	-
136	D	-	F	-	M	-
137	A	-	K	-	K	-
138	K	-	N	n	S	n
139	S	n	T	n	T	G
140	T	n	V	-	V	-
141	V	-	E	-	E	-
142	E	-	I	-	I	-
143	L	-	F	-	F	-
144	F	-	K	-	K	-
145	K	-	F	-	F	-
146	F	-	E	-	E	-
147	Q	-	E	-	E	-
148	E	-	A	-	Q	-
149	E	-	E	-	Q	-
150	E	-	N	n	R	-
151	K	-	S	n	S	n
152	S	n	L	-	L	-
153	L	-	L	-	V	-
154	L	-	H	-	Y	-
155	H	-	L	-	L	-
156	L	-	K	-	K	-
157	K	-	T	G	T	G
158	T	G	V	-	I	-
159	I	-	K	-	K	-

160	R	-	H	-	H	-
161	H	-	E	-	E	-
162	K	-	L	-	L	-
163	L	-	L	-	L	-
164	L	-	P	-	K	-
165	P	-	S	n	S	n
166	N	n	V	-	V	-
167	L	-	N*Cat	n	N*Cat	n
168	N*Cat	n	D*Cat	-	D*Cat	-
169	D*Cat	-	I	-	I	-
170	I	-	T	n	V	-
171	V	-	A	-	V	-
172	A	-	V	-	L	-
173	V	-	G	-	G	-
174	G	-	P	-	P	-
175	P	-	A	-	E	-
176	E	-	H	-	Q	-
177	H	-	F	-	F	-
178	F	-	Y	-	Y	-
179	Y	-	A	-	A	-
180	G	-	T	n	T	n
181	T	n	N	n	R	-
182	N	n	D	-	D	-
183	D	-	H	-	H	-
184	H	-	Y	-	Y	-
185	Y	-	F	-	F	-
186	F	-	S	n	T	n
187	L	-	D	-	N	G
188	D	-	P	-	S	n
189	P	-	F	-	L	-
190	Y	-	L	-	L	-
191	L	-	K	-	S	n
192	Q	-	Y	-	F	-
193	S	n	L	-	F	-
194	W	-	E	-	E	-
195	E	-	T	n	M	-
196	M	-	Y	-	I	-
197	Y	-	L	-	L	-
198	L	-	N	G	D	-
199	G	-	L	-	L	-
200	L	-	H	-	R	-

201	A	-	W	-	W	-
202	W	-	A	-	T	G
203	S	G	N	n	Y	-
204	Y	-	V	-	V	-
205	V	-	V	-	L	-
206	V	-	Y	-	F	-
207	Y	-	Y	-	Y	-
208	Y	-	S	n	S	n
209	S	n	P	-	P	-
210	P	-	N	n	R	-
211	S	n	E	-	E	-
212	E	-	V	-	V	-
213	V	-	K	-	K	-
214	R	-	V	-	V	-
215	V	-	V	-	V	-
216	V	-	A	-	A	-
217	A	-	E	-	K	-
218	E	-	G	-	G	-
219	G	-	F	-	F	-
220	F	-	D	-	C	-
221	D	-	S	n	S	n
222	F	-	A	-	A	-
223	A	-	N*Cat	G	N*Cat	G
224	N*Cat	G	G	-	G	-
225	G	-	I	-	I	-
226	I	-	N	G	T	G
227	N	G	I	-	V	-
228	I	-	S	G	S	G
229	S	n	P	-	A	-
230	P	-	D	-	D	-
231	D	-	D	-	Q	-
232	G	-	K	-	K	-
233	K	-	Y	-	Y	-
234	Y	-	I	-	V	-
235	V	-	Y	-	Y	-
236	Y	-	V	-	V	-
237	I	-	A	-	A	-
238	A	-	D	-	D	-
239	E	-	I	-	V	-
240	L	-	L	-	A	-
241	L	-	A	-	A	-

242	A	-	H	-	K	-
243	H	-	E	-	N	n
244	K	-	I	-	I	-
245	I	-	H	-	H	-
246	H	-	V	-	I	-
247	V	-	L	-	M	-
248	Y	-	E	-	E	-
249	E	-	K	-	K	-
250	K	-	H	-	H	-
251	H	-	T	n	D	-
252	A	-	N	G	N	n
253	N	G (N[1])	M	-	W	-
254	W	-	N	G	D	-
255	T	n	L	-	L	-
256	L	-	T	n	T	n
257	T	n	Q	-	Q	-
258	P	-	L	-	L	-
259	L	-	K	-	K	-
260	K	-	V	-	V	-
261	S	n	L	-	I	-
262	L	-	E	-	Q	-
263	D	-	L	-	L	-
264	F	-	D	-	G	-
265	N	n	T	n	T	G
266	T	n	L	-	L	-
267	L	-	V	-	V	-
268	V	-	D*Cat	-	D*Cat	-
269	D*Cat	-	N*Cat	G (N[1])	N*Cat	G (N[1])
270	N*Cat	G (N[1])	L	-	L	-
271	I	-	S	n	T	G
272	S	G	I	-	V	-
273	V	-	D	-	D	-
274	D	-	P	-	P	-
275	P	-	S	n	A	-
276	E	-	S	n	T	G
277	T	G	G	-	G	-
278	G	-	D	-	D	-
279	D	-	I	-	I	-
280	L	-	W	-	L	-
281	W	-	V	-	A	-
282	V	-	G	-	G	-

283	G	-	C	-	C	-
284	C	-	H	-	H	-
285	H	-	P	-	P	-
286	P	-	N	n	N	n
287	N	n	G	-	P	-
288	G	-	Q	-	M	-
289	M	-	K	-	K	-
290	K	-	L	-	L	-
291	I	-	F	-	L	-
292	F	-	V	-	N	n
293	F	-	Y	-	Y	-
294	Y	-	D	-	N	n
295	D	-	P	-	P	-
296	S	G	N	n	E	-
297	E	-	N	n	D	-
298	N	n	P	-	P	-
299	P	-	P	-	P	-
300	P	-	S	G	G	-
301	A	-	S	G	S	G
302	S	G	E	-	E	-
303	E	-	V	-	V	-
304	V	-	L	-	L	-
305	L	-	R	-	R	-
306	R	-	I	-	I	-
307	I	-	Q	-	Q	-
308	Q	-	N	G	N	n
309	N	n	I	-	V	-
310	I	-	L	-	L	-
311	L	-	S	n	S	n
312	T	n	E	-	E	-
313	E	-	K	-	K	-
314	E	-	P	-	P	-
315	P	-	T	G	R	-
316	K	-	V	-	V	-
317	V	-	T	n	S	G
318	T	G	T	G	T	G
319	Q	-	V	-	V	-
320	V	-	Y	-	Y	-
321	Y	-	A	-	A	-
322	A	-	N	n	N	n
323	E	-	N	G (N[1])	N	G (N[1])

324	N	G (N[1])	G	-	G	-
325	G	-	S	n	S	n
326	T	G	V	-	V	-
327	V	-	L	-	L	-
328	L	-	Q	-	Q	-
329	Q	-	G	-	G	-
330	G	-	S	n	T	G
331	S	G	S	G	S	G
332	T	G	V	-	V	-
333	V	-	A	-	A	-
334	A	-	S	n	S	n
335	S	n	V	-	V	-
336	V	-	Y	-	Y	-
337	Y	-	D	-	H	-
338	K	-	G	-	G	-
339	G	-	K	-	K	-
340	K	-	L	-	I	-
341	L	-	L	-	L	-
342	L	-	I	-	I	-
343	I	-	G	-	G	-
344	G	-	T	n	T	n
345	T	n	L	-	V	-
346	V	-	Y	-	F	-
347	F	-	H	-	H	-
348	H	-	R	-	K	-
349	K	-	A	-	T	n
350	A	-	L	-	L	-
351	L	-	Y	-	Y	-
352	Y	-	C	-	C	-
353	C	-	E	-	E	-
354	E	-	L	-	L	-
355	L	-				
Glycosylation percent	7%		6%		7%	

Predicted glycosylation patters of PON1 (PMID Accession no. P27169), PON2 (PMID Accession no. Q15165), and PON3 (PMID Accession no. Q15166) through computational modeling and analysis obtained from (https://comp.chem.nottingham.ac.uk/cgi-bin/glyco/bin/getparams.cgi); *Cat Denotes Amino acid residue in catalytic region; G = Predicted glycosylated residue; n = Residue is not predicted to be glycosylated; - = Other residue; N[1]=published N-linked glycosylation residue
